# Visualization of the Duct of Luschka During a Robotic-Assisted, Laparoscopic Cholecystectomy: A Report of a Rare Event

**DOI:** 10.7759/cureus.84756

**Published:** 2025-05-24

**Authors:** Gerard V Giangrosso, Curtis W Harrison, David Denning, Farzad Amiri

**Affiliations:** 1 General Surgery, Marshall University Joan C. Edwards School of Medicine, Huntington, USA

**Keywords:** duct of lushka, post-cholecystectomy bile leak, robotic assisted cholecystectomy, robotic surgical procedures, subvesical bile duct

## Abstract

A laparoscopic cholecystectomy is one of the most common surgeries performed by general surgeons in the United States. With the increasing amount of robotic surgery being performed, there are clinical scenarios seen from entirely new angles, given the improved visualization that the da Vinci Surgical System (Intuitive Surgical, Inc., Sunnyvale, California, United States) offers. One of these potential scenarios is the visualization of subvesical ducts, or better known as the duct of Luschka. In this case, we were able to use the da Vinci to clearly visualize one of these ducts in the process of getting our critical view of safety, and control it without difficulty. This report provides an example to discuss the management of ducts of Luschka and their bile leaks, if noted during the initial operation.

## Introduction

A laparoscopic cholecystectomy is one of the most common surgeries performed by general surgeons in the United States, with around 600,000 being performed annually [1]. Over the past several years, the procedure has evolved to include the use of the da Vinci Xi Robotic System (Intuitive Surgical, Inc., Sunnyvale, California, United States), and many surgeons are starting to incorporate it into their practice. Aguayo et al., in 2020, showed that rates of robotic-assisted, laparoscopic cholecystectomy were increasing, from 0.02% in 2008 to 3.2% of all laparoscopic cholecystectomies in 2017 [2]. Residencies are also starting to include robotic surgery in the curriculum, meaning that this number will only increase in the coming years.

With the increasing number of robotic surgeries being performed, there will be complications and clinical scenarios seen from entirely new angles, given the improved visualization that the da Vinci offers. One of these potential scenarios is the visualization of a subvesical duct, better known as a duct of Luschka. Present in approximately 4-10% of the population, a leak from one of these ducts occurs in 0.2-2% of all laparoscopic cholecystectomies, making it the second most common cause of a bile leak [3,4]. Many times, due to the size of these ducts, they cannot be visualized intraoperatively even with the laparoscope, and many times, a leak is not discovered until it occurs postoperatively on an endoscopic retrograde cholangiopancreatography (ERCP). The incidence of these ducts being visualized during surgery is unknown. Some prior studies have also noted that a duct of Luschka is a particular anatomical variation of concern, on par with cystic duct anomalies and cystic artery anomalies [5].

In this report, we describe a case in which we were able to use the da Vinci to clearly visualize one of these ducts in the process of getting our critical view of safety during a cholecystectomy. Once we confirmed that this was a subvesical duct, it was easily addressed with no postoperative issues. This case serves as an example to discuss the management of the ducts of Luschka and their bile leaks, if noted during the initial operation. 

This case was earlier presented as a poster at the 2025 Southeastern Surgical Congress (SESC) Annual Meeting.

## Case presentation

 A 49-year-old female patient presented with right flank pain. The pain had occurred intermittently for a few months and was described as a cramping pain. She was unsure if the pain was associated with meals, and denied nausea and vomiting. She had no prior medical or surgical history. On exam, she was mildly tender to palpation, but had a negative Murphy sign. A CT scan ordered by her primary care physician showed gallstones, but no obvious signs of acute cholecystitis. At this point, a diagnosis of symptomatic cholelithiasis was made, and the patient consented to a robotic-assisted, laparoscopic cholecystectomy.

The patient was brought to the operating theater and placed in the supine position. She was induced under endotracheal anesthesia and then prepped and draped in the usual sterile fashion. A Veress needle was inserted at Palmer’s point, and the abdomen was insufflated to 15 mmHg. An 8 mm robotic trochar was placed in the left upper quadrant, and after visualizing no injuries, the Veress needle was removed. Two more 8 mm trochars and one 12 mm trochar were placed in a transverse abdominal plane. The patient was placed head up, left side down, and the robot was docked.

**Figure 1:**
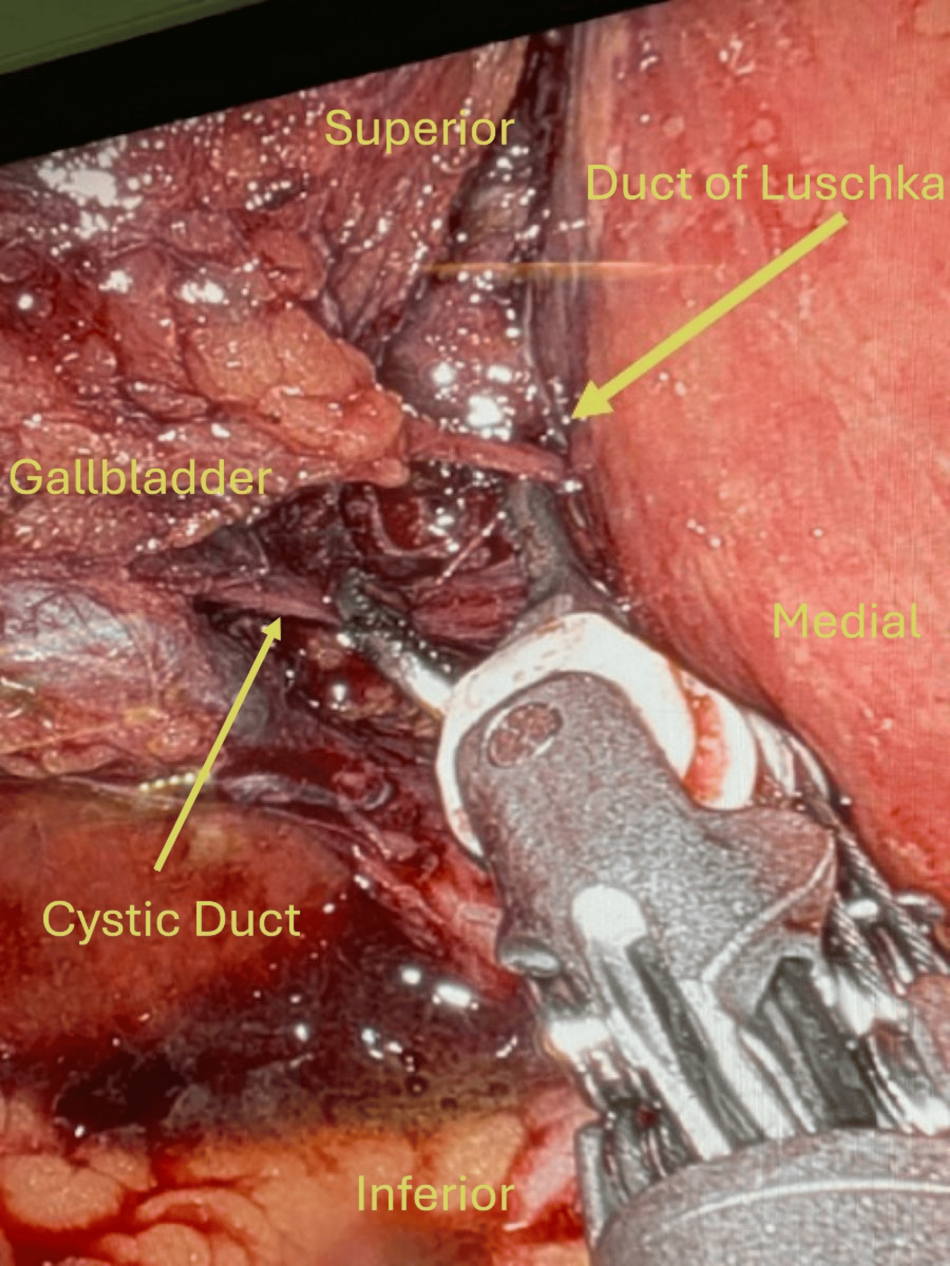
Visualized Duct of Luschka

**Figure 2:**
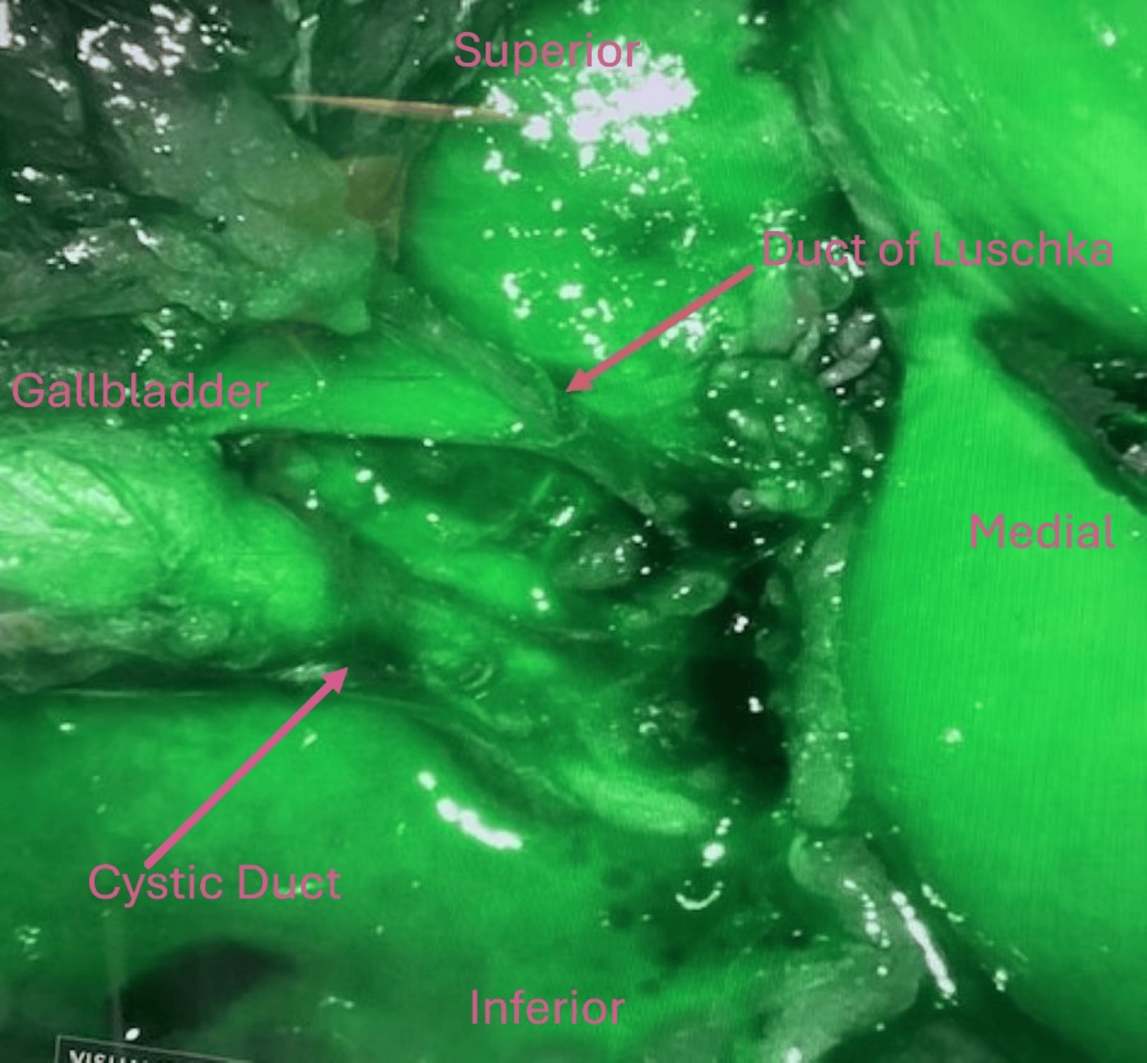
Indocyanine green (ICG) Entering Duct of Luschka

The gallbladder was visualized and retracted cephalad above the liver. Once the infundibulum was identified, the peritoneum was taken down, and the cystic duct and artery were dissected to achieve the critical view of safety. At this point, an accessory duct was clearly visualized (Figure 1) in addition to the cystic duct and artery. This was confirmed to be a duct of Luschka via the use of Indocyanine green (ICG) dye angiography, as it showed ICG entering the gallbladder like the cystic duct (Figure 2). There were no signs of allergic reaction or anaphylaxis after administration. The rest of the liver bed was then dissected, and no other additional aberrant or accessory anatomy was noted. All three structures were clipped proximal and distal and then cut. The gallbladder was then removed from the liver using scissor electrocautery. Hemostasis was achieved, and the gallbladder was removed from the abdomen using an Endo Catch™ Specimen Retrieval Pouch (Medtronic plc, Galway, Ireland) through the 12 mm port site. The 12 mm port site fascia was closed using an EndoClose device, and the skin was then closed with Monocryl (Ethicon Inc., Raritan, New Jersey, United States) and Dermabond (Ethicon Inc.). The patient was extubated and brought to the Post Anesthesia Care Unit (PACU) in stable condition. Postoperatively, she did well with no complications on the postoperative visit.

## Discussion

This report documents a rare instance of being able to visualize a duct of Luschka during surgery and addressing it before it becomes a problem postoperatively. Many times, no duct is ever visualized and is only discovered after a patient returns with a bile leak. As such, younger surgeons may pause when seeing these ducts during surgery and wonder how they are managed. We seek to further discuss their management.

As noted above, subvesical ducts or ducts of Luschka are a collection of variable anatomical variations in biliary drainage [4]. They can be essentially broken down into four types: segmental/sectorial, accessory, hepatiocholecystic, and/or aberrant bile ducts. Most often, they drain into the right hepatic duct or the common hepatic duct, and surprisingly, not into the gallbladder [4,6]. While there is no consensus on their origin, there are two hypotheses: the first is that these ducts are congenital and form during the third or fourth week of gestation [7]. The other is that these ducts form due to tissue remodeling from inflammation, causing hypertrophy of the hepatic bile ducts [4]. Embryologically, there is also no consensus as to their origin, as the anatomic variation makes it difficult to determine.

Regardless of how they form, most surgeons are concerned about their tendency to cause a postoperative bile leak. A leak from one of these subvesical ducts is only second to cystic duct leak as the most common cause of bile leak postoperatively and is typically included in the Strasberg Classification as a type A injury [3]. If one does occur, patients tend to revisit during the first postoperative week with complaints of continued abdominal pain, nausea/vomiting, and general malaise. The established workup includes a complete metabolic panel and a combination of a right upper quadrant ultrasound, CT abdomen/pelvis, and a hepatobiliary iminodiacetic acid (HIDA) scan. Typically, bilirubin will be elevated, and the ultrasound and CT scan will visualize a fluid collection in the gallbladder fossa. A HIDA scan can also show extravasation, worrisome for a bile leak. After a percutaneous drain is placed to control the biloma, an ERCP is performed for both diagnostic and therapeutic options [8]. The endoscopist can use the contrast to localize the location of the leak and treat it appropriately. If it is truly a duct of Luschka leak, at the time of the ERCP, typically a biliary duct stent and endoscopic sphincterotomy are performed as well [8]. This allows for the reduction of intraductal pressure and encourages the flow of bile through the common bile duct, as opposed to the duct of Luschka [8]. The duct can heal once bile flow decreases through it. Rarely, if endoscopic management fails, reoperation via laparoscopy to directly ligate the duct is the next escalation of therapy. There are older case reports of laparotomies being performed to control bile leakage, but this seems to have decreased significantly with the increasing skills and technology afforded to endoscopists [9].

After a thorough literature review, there seems to be no definitive guidelines on the management of subvesical ducts intraoperatively. However, there are several case reports of various management techniques. One case report by Spanos and Spanos described a subvesical bile duct visualized during a laparoscopic cholecystectomy before gallbladder removal, and it was controlled via clips and ligated in a manner like the cystic duct [10]. This was how it was controlled during our case, too. Kohga et al. managed these ducts slightly differently [11]. They did not visualize the ducts until after the gallbladder was removed and a small amount of bile was seen leaking from the gallbladder fossa. The ducts were then closed using suture ligation. Masoni et al. also visualized a leaking duct after gallbladder removal in the fossa and controlled it using clips [12]. None of the cases had any issue with a symptomatic bile leak postoperatively. Clearly, it is advantageous to visualize the ducts and ligate them before they leak, but should an intraoperative leak be visualized, suture ligation and clips both seem to work well.

## Conclusions

As robotic surgery increases, visualization and intraoperative management of the ducts of Luschka will only increase in frequency. When visualized prior to gallbladder removal, these ducts can safely be managed with clips and ligation, like the cystic duct and cystic artery, without concerns for postoperative leaks. If they are not noticed until after gallbladder removal, clips and/or suture ligation also seem to control any bile leakage.
